# An Emergency Packet Forwarding Scheme for V2V Communication Networks

**DOI:** 10.1155/2014/480435

**Published:** 2014-06-25

**Authors:** Faika Hoque, Sungoh Kwon

**Affiliations:** School of Electrical Engineering, University of Ulsan, Ulsan 680-749, Republic of Korea

## Abstract

This paper proposes an effective warning message forwarding scheme for cooperative collision avoidance. In an emergency situation, an emergency-detecting vehicle warns the neighbor vehicles via an emergency warning message. Since the transmission range is limited, the warning message is broadcast in a multihop manner. Broadcast packets lead two challenges to forward the warning message in the vehicular network: redundancy of warning messages and competition with nonemergency transmissions. In this paper, we study and address the two major challenges to achieve low latency in delivery of the warning message. To reduce the intervehicle latency and end-to-end latency, which cause chain collisions, we propose a two-way intelligent broadcasting method with an adaptable distance-dependent backoff algorithm. Considering locations of vehicles, the proposed algorithm controls the broadcast of a warning message to reduce redundant EWM messages and adaptively chooses the contention window to compete with nonemergency transmission. Via simulations, we show that our proposed algorithm reduces the probability of rear-end crashes by 70% compared to previous algorithms by reducing the intervehicle delay. We also show that the end-to-end propagation delay of the warning message is reduced by 55%.

## 1. Introduction

According to the National Center for Statistics and Analysis (NCSA) of the National Highway Traffic Safety Administration (NHTSA), vehicle crashes are the leading cause of death in the United States [1]. Rear-end collisions account for almost one-third of all traffic crashes [2]. Driver inattention is a major factor in 91% of rear-end crashes, as reported in [3]. Most of the rear-end collisions resulted from insufficient time for drivers to react in an emergency situation. A driver typically depends on the visual information of the immediately preceding vehicle. In road emergency situations, if a vehicle does not notice the visual information in time or there is not enough given time to brake or react, a chain collision could result. According to [4], an extra 0.5 seconds of warning time can prevent about 60% of rear-end collisions.

The following simplified example illustrates a chain collision due to inadequate time to react in an emergency situation. We assume that three vehicles are travelling in the same lane at the identical speed of 110 km/hr and are spaced by 30 m. It is also assumed that the vehicles decelerate at 4 m/s^2^ and follow the visual information of the immediately preceding vehicle. After observing an emergency situation, the first vehicle brakes abruptly. In general, driver reaction time (the duration time between when an event is observed and when the driver brakes) is in the range of 0.75 s to 1.5 s [5]. Suppose that the second vehicle takes 0.75 s and the third vehicle takes 1.5 s to react; then although the second vehicle can stop before colliding with first vehicle, the third vehicle crashes into the second vehicle.

The intelligent transportation system (ITS) can help drivers to react in an emergency situation. ITS provides a framework to alleviate traffic congestion and improves public safety goals such as collision avoidance. The allocation of 75 MHz in the 5.2 GHz band for licensed dedicated short range communication (DSRC) delivers high media contents to vehicle-to-vehicle (v2v) communications [6, 7]. To enhance highway public safety, a cooperative collision avoidance (CCA) system can be used via v2v wireless communication [8]. In such cooperative systems, vehicles determine the emergency situation and make a decision whether to warn others based on the information given by the neighbor vehicles.

As soon as an emergency situation is detected, a vehicle warns its neighboring vehicles via an emergency warning message (EWM), and the recipients of the warning message announce the warning to their neighbors. Since acknowledgement of one-to-one transmission produces more delay and all the neighbors should be aware of the warning situation immediately, EWMs are broadcast to neighbors [9, 10]. Moreover, to improve the delivery success, EWMs are periodically rebroadcast.

Although such cooperative v2v communication systems can improve highway safety by using emergency warning messages, reliable transmissions and stringent delay are the main requirements to deliver a warning message in an emergency situation. If the EWM delivery latency is large, then a chain collision will occur. Therefore, an efficient EWM forwarding protocol is necessary to reduce redundant broadcasting and packet collisions, which impede the warning message delivery, at the media access control (MAC) layer.

Previous work has taken effort to reduce the delivery latency of EWM by improving the warning message forwarding protocol and MAC enhancement. To reduce redundant broadcasting, intelligent broadcasting with implicit acknowledgement (I-BIA) is proposed in [9, 10]. In this proposed method, implicit acknowledgement is adopted to reduce redundant broadcasting, in which reception of the duplicate EWM acts as implicit acknowledgment. After receiving a duplicate EWM from one of vehicles behind itself, the vehicle stops broadcasting the EWM packet. Note that I-BIA uses only one-way implicit acknowledgement to stop the redundant broadcasting assuming that all vehicles in the vehicle chain received the warning messages. If an intermediate vehicle in the chain cannot receive the warning message due to transmission collisions, the proposed algorithm delays the recognition of emergency at the vehicle until an EWM arrives from a preceding vehicle. If the intermediate vehicle receives the EWM after more than 500 ms delay, then a chain collision will occur [4]. The algorithm also ignores the warning message delay in the dense network. Moreover, the algorithm uses only binary exponential back-off (BEB) for EWM. Binary exponential back-off induces a large delay to propagate the warning message as the senders enter into a long inhibition period before transmission.

To reduce the EMW propagation delay due to exponential random back-off, a fixed contention window (CW) is proposed in [11] with implicit acknowledgment. While a fixed contention window induces a small delay in a sparse network, it causes an extremely large delay in a dense network due to the large number of transmissions among EWMs. An adaptable offset slot (AOS) mechanism is proposed in [12] to reduce the delay by initializing different contention window sizes depending on the number of neighbor vehicles. Since the algorithm linearly increases the contention window as the number of vehicles increases, an emergency message has a large delay to overcome the nonemergency transmissions in a dense network.

To overcome nonemergency transmissions, an adaptable distance-dependent random back-off algorithm is proposed in [13]. The algorithm adopts heterogeneous random back-off based on the location and the number of neighbor vehicles. However, the proposed algorithm has no scheme to reduce the redundant EWMs, which results in collisions among EWMs and induces delay in propagation of the warning message.

In this paper, we propose a two-way intelligent broadcasting scheme to reduce the redundant broadcasting of EWMs and avoid a chain collision among intermediate vehicles. Moreover, a composite random back-off is adopted for EWM to compete with nonemergency transmissions and emergency transmissions in a dense network. The performance is evaluated via simulations in different environments.

The rest of this paper is organized as follows. In Section 2, we describe the system model and problem of the IEEE 802.11 DCF-based MAC protocol for EWM in a vehicle environment. In Section 3, we propose an efficient forwarding strategy with an adaptable random back-off for EWM transmission. In Section 4, we analyze the performance of the proposed algorithm via simulation results. Finally, we conclude the paper in Section 5.

## 2. System Model and Challenge

### 2.1. System Model

All vehicles on the highway are assumed to be equipped with wireless communication devices based on the IEEE 802.11p standard. The IEEE 802.11p uses the basic mechanism of the distributed control function (DCF) as the fundamental protocol for medium access control. The protocol adopts the carrier sense multiple access with collision avoidance (CSMA/CA) as a random access scheme for all vehicles [14]. We also assume that all vehicles are equipped with global positioning system (GPS) [9, 15] to estimate location. In a normal situation, vehicles establish a mobile ad hoc network via a routing protocol and exchange nonemergency data with each other.

When noticing an emergency situation, an event-detecting vehicle broadcasts an EWM to immediately warn the neighbor vehicles. Due to limited transmission range, all vehicles receiving the warning message relay the message to neighbor vehicles, which then forward the message in a multihop manner. Before transmitting the EWM, vehicles sense the medium to check whether it is idle or busy via CSMA/CA scheme [16]. If the medium is idle during interframe space (IFS), the scheme waits for a random back-off period time before transmitting data. A random back-off period is a waiting slot time, which is randomly chosen between 0 and the contention window (CW), before transmitting the packet to avoid the collision. The back-off time is decreased from the randomly chosen time as long as the medium is sensed to be idle. When the scheme reaches time 0, it transmits the packet. If the medium is busy, the vehicle defers message transmission until the end of the current transmission, and the CSMA/CA procedure starts again. Since broadcasting does not support acknowledgement, to improve delivery efficiency, EWM is periodically rebroadcast.

In such CSMA/CA-based v2v networks, redundant EWMs induce high collisions to access the channel. Moreover, EWMs compete with two categories of transmission: EWMs and nonemergency transmissions. In next subsection, we discuss two major challenges in a v2v network in detail and propose an effective EWM forwarding scheme in the following section.

### 2.2. Challenges in Transmitting EWM

#### 2.2.1. Redundant Broadcast of EWM

Since EWM is periodically broadcast to improve the delivery success rate, vehicles which have already successfully forwarded the message to the vehicles behind will keep broadcasting the message. Moreover, overlapping transmission ranges of vehicles also induce collision among EWMs. The primary limitation of this network is a broadcasting storm in the network, which results in packet collisions for channel access and a hindrance of EWM forwarding to neighboring vehicles. The effect of redundant EWMs may be serious hazard situations in emergency cases.

**Figure 1:**
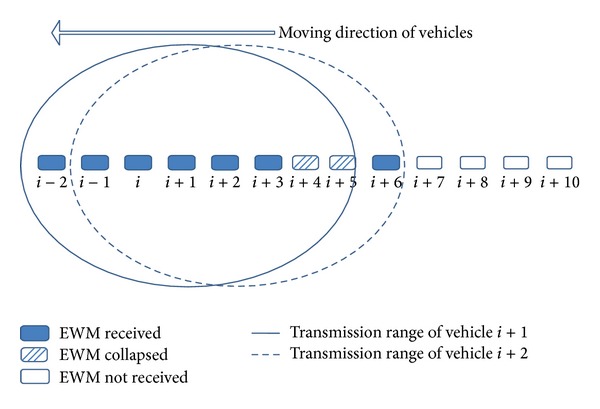
EWM collisions in the vehicle chain.

For example, let us assume that an emergency-detecting vehicle broadcasts an EWM to warn the neighbors of an emergency and that all vehicles up to *i* + 3 received the warning message in a multihop manner, as shown in Figure 1. If vehicles *i* + 1 and *i* + 2 transmit the message after the random back-off, vehicle *i* + 6 receives the message from vehicle *i* + 2, but due to EWM transmission collision, vehicles *i* + 4 and *i* + 5 do not receive the warning message. Such EWM-unaware vehicles in the chain have greater possibility for rear-end crashes.

#### 2.2.2. Competition with Nonemergency Messages

**Figure 2:**
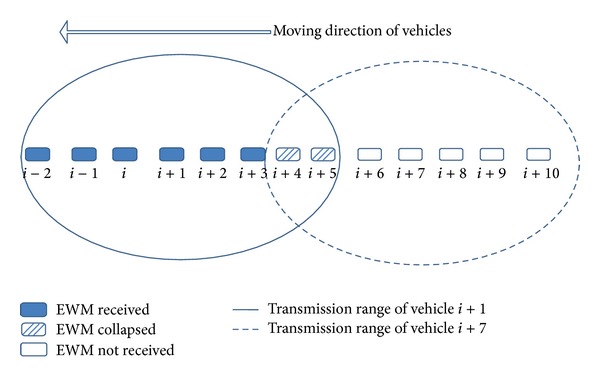
Interference of EWM with nonemergency transmissions.

Vehicles in the chain can be categorized into two groups: vehicles that did not receive the EWM and vehicles that received the EWM, as shown in Figure 2. Vehicles which did not receive the warning message due to limited transmission range are unaware of the emergency situation and continue to send their existing nonemergency messages. The vehicles that received the warning message relay the message by periodically rebroadcasting. However, nonemergency transmissions prohibit further warning message forwarding. Therefore, vehicles close to the boundary region, such as vehicles *i* + 2 and *i* + 3 in Figure 2, are more affected by nonemergency transmissions. If vehicles that did not receive EWM are present among vehicles receiving the EWM, as in Figure 1, the interference with nonemergency transmissions will significantly affect the EWM forwarding. Hence, the EWM-unaware vehicles among EWM-aware vehicles not only are in a hazardous situation due to EWM unawareness, but also jeopardize other vehicles due to hindrance to disseminate the EWM.

Vehicles receiving EWM should have a small contention window for EWM to compete with nonemergency transmissions, although these same vehicles should contradictorily have a large contention window to reduce collisions among EWM transmissions.

## 3. Proposed Algorithm

In this section, two-way intelligent broadcasting with implicit acknowledgement (2I-BIA) is proposed to reduce redundant EWMs where vehicles receive the acknowledgement from both directions. In addition, to compete with nonemergency transmissions, vehicles close to the boundary region use a small contention window. To reduce the collisions among EWMs, vehicles in the near region of the corresponding EWM sender use a conventional contention window.

To that end, we employ the EWM format proposed in [10]. The EWM contains an original vehicle ID, an event ID, a message type, and a sender's location. The origin vehicle ID and the event ID uniquely identify a message across the network. The message type informs others of an emergency situation. With the sender's location and the receiver's location, the receiver recognizes the direction of the message, from a preceding vehicle or a following vehicle, and measures the distance between the sender and itself.

When receiving an EWM, a vehicle decides if the EWM is a new message based on the originating vehicle ID and the event ID. If the EWM is a new message, the vehicle stores the message and periodically rebroadcasts the message with an adaptable contention window, which will be described later.

If the EWM is a duplicate, the vehicle determines whether the message is from a sender that is in the opposite direction of the previous EWM sender. For example, the previous EWM came from a vehicle that is behind (or in front of) the receiver, and the present EWM arrived from a vehicle that is in front of (or behind) the receiver. In such a case, the EWM-receiving vehicle recognizes the EWM as an implicit acknowledgement of the previous EWM so that the vehicle stops periodic broadcasting of the EWM. If the duplicate EWM comes from a vehicle that is in the same direction as the previous EWM sender with respect to the receiver, then the receiver keeps periodically broadcasting the EWM with an adaptable contention window.

When a vehicle periodically broadcasts an EWM, in order to compete with nonemergency transmissions and reduce the possibility of collisions among EWMs, an adaptable contention window is employed, which is introduced in [13]. To employ an adaptable contention window, a threshold value is chosen as a function of neighbor vehicles and contention window size. If a vehicle receiving an EWM is close to vehicles that do not receive an EWM, the vehicle chooses a short and fixed contention window size for random back-off. Otherwise, an ordinary binary random back-off is used. In other words, if a receiver is behind the corresponding EWM sender with respect to the driving direction and the distance from the sender to the receiver is greater than a threshold, the receiver is determined to be in the area of competition with nonemergency messages and chooses a fixed small contention window to compete. Otherwise, assuming that the collisions among EWMs are more dominant, an ordinary binary random back-off is chosen. The algorithm is summarized in Algorithm 1.

## 4. Simulations

The proposed system is implemented using the OPNET 16.0 network simulator [17]. The simulation results are the average of 20 runs using different random seeds unless stated otherwise. The physical characteristics follow the specifications of 802.11 with 11 MHz of bandwidth. The transmission range of each vehicle is 300 m, as specified by the DSRC [6, 18]. The underlying MAC protocol is based on the 802.11 DCF function. To employ the proposed adaptable random back-off algorithm for the EWM, the value of the CW_fix_ is set to 7 for the fixed random back-off, and the values of the CW_min⁡_ and CW_max⁡_ are set to 15 and 1023, respectively, for the ordinary binary random back-off algorithm. We consider nonemergency transmissions such as communications between vehicles and control messages for routing [9, 11].

For simplicity, we assume that vehicles are driving in one direction in a single lane. To maintain a safe distance between vehicles, the intervehicle spaces are based on the driving speed. If there is no traffic congestion, vehicles move at high speed, and the intervehicle space will be large. If traffic congestion is present, vehicles move slowly and have a high density. Hence, we assume that the vehicles are uniformly distributed over 1 km and that the number of vehicles ranges from 20 to 140.

When an emergency event occurs, the event-detecting vehicle broadcasts an EWM packet to warn the neighbor vehicles. The length of the packet is 1024 bits. Since the vehicle does not wait for acknowledgement, the EWM is rebroadcast every 50 ms to ensure successful transmission. After receiving the warning message, the neighboring vehicles rebroadcast the packet until it reaches the end of the vehicle chain. In this way, drivers become aware of the emergency situation and start to decelerate to avoid the collision.

For performance measurement, we consider intervehicle delay and end-to-end delay. The intervehicle delay is the time difference of receiving the EWM between two adjacent vehicles. The end-to-end delay is defined as the elapsed time from when the first vehicle transmits an EWM to the time when the last vehicle in the lane receives the EWM. We compare the performance of our proposed algorithm 2I-BIA with the original CSMA/CA, I-BIA proposed in [9] and adaptable distance-dependent back-off algorithm (ADDB) proposed in [13].

### 4.1. Intervehicle Delay

In this section, we study the receiving time of an EWM at each vehicle after the first emergency-detecting vehicle begins broadcasting. If the receiving time of an EWM between two adjacent vehicles is large, then a chain collision may occur because the vehicles will not have enough time to brake or react to the emergency situation.

**Figure 3:**
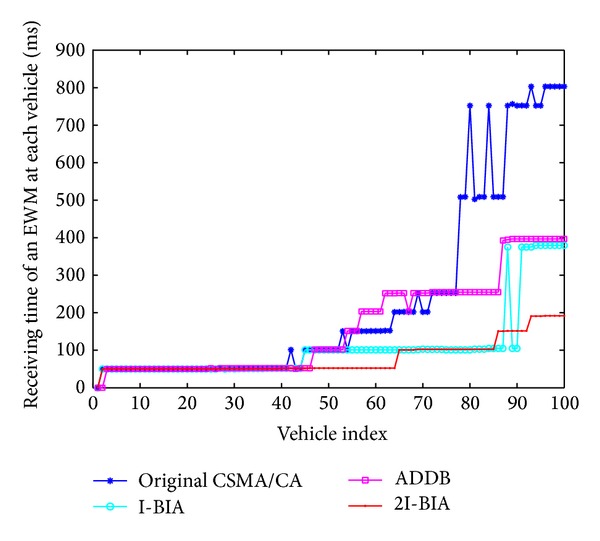
Receiving time of first EWM at each vehicle.

Figure 3 shows the receiving time of an EWM packet at each vehicle when the first of 100 vehicles initiates the warning message in the presence of 100 kbps nonemergency message traffic. Up to the 40th vehicle, the receiving time is almost the same at each vehicle. However, in the region far from the first vehicle, when the number of transmissions increases, the receiving times at each vehicle are different due to transmission collisions among redundant EWMs and competition of the EWM with the nonemergency transmissions. In the case of the original CSMA/CA, the receiving time is abruptly increased between the 79th vehicle and the 80th vehicle, and the 80th vehicle produces a discontinuity between the 79th and 81th vehicles. Although I-BIA and ADDB reduce the intervehicle delay, discontinuity between adjacent vehicles in a vehicle chain still occurs. However, our proposed 2I-BIA algorithm reduces the probability of discontinuity in the vehicle chain by reducing the intervehicle delay of adjacent vehicles. Since the proposed two-way acknowledgement reduces the redundant EWMs and ensures successful delivery in both directions, all vehicles in the chain receive the EWM after less than 100 ms intervehicle delay.

**Figure 4:**
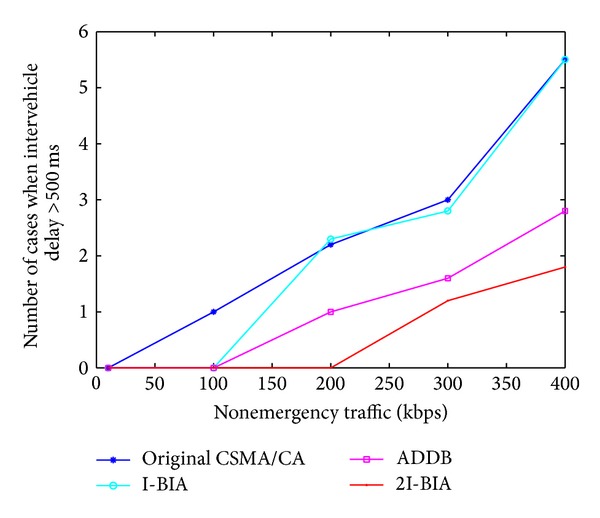
Number of cases where intervehicle delay is ≥500 ms.

Greater intervehicle delay induces a higher possibility of chain collision. According to [4], if driver has an extra 500 ms to react in an emergency situation, 60% of chain collisions are avoidable. Hence, we discuss the number of cases in which intervehicle delays are greater than 500 ms.  Figure 4 shows the number of cases when intervehicle delay is greater than 500 ms with the presence of different levels of nonemergency transmissions. Simulations are performed with 100 vehicles in the presence of different levels of nonemergency transmissions. Simulation results show that, with other algorithms, at least one vehicle experiences a 500 ms intervehicle delay when the nonemergency traffic is 200 kbps, while our proposed algorithm gives an intervehicle delay less than 500 ms. As the number of nonemergency transmissions increases, the number of cases where intervehicle delay is greater than 500 ms also increases. However, our proposed algorithm reduces the collision probability by at least 70% compared with the I-BIA algorithm.

### 4.2. End-To-End Delay

**Figure 5:**
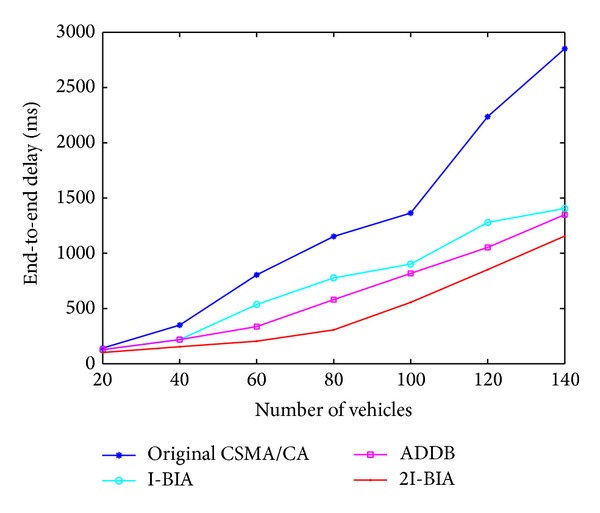
End-to-end delay of an EWM packet with various numbers of vehicles.

In this section, we compare the average end-to-end delay as performance measurement in the presence of nonemergency traffic as the number of vehicles varies.  Figure 5 shows the end-to-end delay with 200 kbps nonemergency traffic. As the number of vehicles increases, the number of retransmissions also increases, which results in more delay in all the algorithms. However, our proposed algorithm uses short CW to overcome nonemergency transmissions and two-way acknowledgement to reduce redundant EWMs to produce a shorter delay than the other methods.

**Figure 6:**
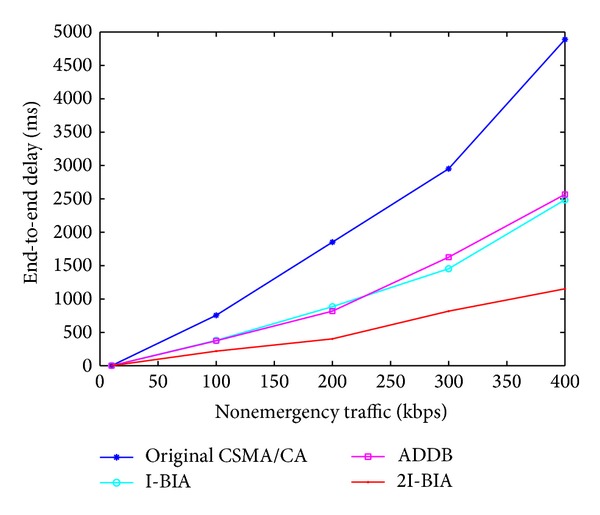
End-to-end delay of an EWM packet with different nonemergency traffic levels.

As nonemergency transmissions increase, the end-to-end delay also increases, as shown in Figure 6. For all cases, our algorithm outperforms the other algorithms and improves the performance by approximately 55% compared with I-BIA and ADDB.

## 5. Conclusions

In this paper, we proposed an emergency warning message forwarding scheme to improve message delivery efficacy in vehicular environments. In a v2v network, two challenges prevent warning message forwarding: redundant EWM transmissions from EWM recipients and nonemergency transmissions from the emergency-unaware vehicles. To overcome these challenges, the proposed algorithm intelligently broadcasts emergency warning messages using duplicates as implicit acknowledgements and adopting an adaptable contention window size. If a vehicle is recognized to be between EWM recipients based on duplicate EWMs, then the vehicle stops relaying the EWM. Moreover, a vehicle broadcasts EWM using heterogeneous contention window sizes to reduce EWM transmission collision and compete with nonemergency transmissions. Simulation results show that our proposed algorithm reduces the intervehicle delay by 70% and the end-to-end delay by 55% compared to previous algorithms, thus reducing the possibility of a chain collision.

## Figures and Tables

**Algorithm 1:**
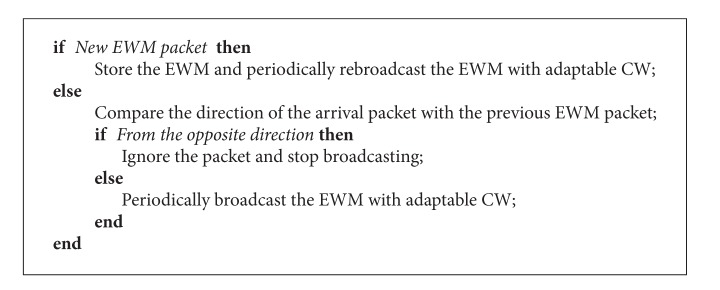
Two-way intelligent broadcast with implicit acknowledgment (2I-BIA).
